# Advances in Plant Sulfur Research

**DOI:** 10.3390/plants9020256

**Published:** 2020-02-17

**Authors:** Dimitris L. Bouranis, Mario Malagoli, Jean-Christophe Avice, Elke Bloem

**Affiliations:** 1 Plant Physiology Laboratory, Crop Science Department, Agricultural University of Athens, Iera Odos 75, 11855 Athens, Greece; 2 Department of Agronomy, Food, Natural Resources, Animals and Environment, University of Padova, Agripolis, 35020 Legnaro Pd, Italy; mario.malagoli@unipd.it; 3 UMR INRA-UCN 950 Ecophysiologie Végétale, Agronomie & Nutritions N.C.S., Normandie Université, UFR des Sciences, FED 4277 Normandie Végétal, Université de Caen Normandie, F-14032 Caen, France; jean-christophe.avice@unicaen.fr; 4 Institute for Crop and Soil Science, Julius Kühn-Institut (JKI), Federal Research Centre for Cultivated Plants, Bundesallee 69 (Gebäude 250), D-38116 Braunschweig, Germany; elke.bloem@julius-kuehn.de

**Keywords:** sulfur deficiency, sulfur nutrition physiology, sulfur interactions

## Abstract

As an essential nutrient required for plant growth and development, sulfur (S) deficiency in productive systems limits yield and quality. This special issue hosts a collection of original research articles, mainly based on contributions from the 11th International Plant Sulfur Workshop held on 16–20 September 2018 in Conegliano, Italy, focusing on the following topics: (1) The germinative and post-germinative behaviour of *Brassica napus* seeds when severe S limitation is applied to the parent plants; (2) the independence of S deficiency from the mRNA degradation initiation enzyme PARN in Arabidopsis; (3) the glucosinolate distribution in the aerial parts of sel1-10, a disruption mutant of the sulfate transporter SULTR1;2, in mature *Arabidopsis thaliana* plants; (4) the accumulation of S-methylcysteine as its γ-glutamyl dipeptide in *Phaseolus vulgaris*; and (5) the role of ferric iron chelation-strategy components in the leaves and roots of maize, have provided new insights into the effect of S availability on plant functionality. Moreover, the role of S deficiency in root system functionality has been highlighted, focusing on (6) the contribution of root hair development to sulfate uptake in Arabidopsis, and (7) the modulation of lateral root development by the CLE-CLAVATA1 signaling pathway under S deficiency. The role of S in plants grown under drought conditions has been investigated in more detail focusing (8) on the relationship between S-induced stomata closure and the canonical ABA signal transduction machinery. Furthermore, (9) the assessment of S deficiency under field conditions by single measurements of sulfur, chloride, and phosphorus in mature leaves, (10) the effect of fertilizers enriched with elemental S on durum wheat yield, and (11,12) the impact of elemental S on the rhizospheric bacteria of durum wheat contributed to enhance the scientific knowledge on S nutrition under field conditions.

Sulfur (S) is an essential nutrient required for plant growth and development. The environmental protection measures put in place in the last decades and the reduction of SO_2_ emission to the atmosphere paradoxically limited the availability of S as input in large scale agriculture with consequent crop S deficiency. Therefore, S containing fertilizers are nowadays used worldwide to support enhancing crop yield and quality. The emergence of S deficiency in such productive systems attracted the scientific attention and triggered significant research interest. In the last 25 years a tremendous boost has taken place in all aspects of plant S research. Milestones in this field include so far the central role of sulfate transporters in response to S availability, sulfur as a part of plant metabolic network, sulfotranferases activity, impact of S on N_2_ fixation of legumes, role of S compounds in abiotic and biotic stress tolerance, S interactions in crop ecosystems, S-induced-resistance, molecular links between metals in the environment and plant S metabolism, role of sulfate and S-rich compounds in heavy metal tolerance and accumulation.

Despite the amazing amount of the rapidly accumulating information, there are still open questions and challenges on this fascinating field of research. Sulfur has unique characteristics in various aspects, some of which provide potential areas for practical applications, as summarized by Kopriva et al. (2019) [1], pointing out that still significant gaps in our knowledge remain in several aspects. For example, the effects of the availability of other mineral nutrients on S utilization need to be studied more in detail by focusing on their interactions. The functions of S metabolites in assisting mutualistic interactions between living organisms (i.e., microbes, animals, and plants) are not fully understood, while with the exception of a number of known cases characterized so far for essential biological or nutritional functions, the diversity of S metabolites appears to be an unexplored area worth further studies. Moreover, given the importance of the geochemical cycle of S in nature, the roles of S nutrition and S-containing metabolites in the environmental adaptation of plants need to be studied with more attention given to eco-physiological aspects.

This special issue hosts a collection of papers based mainly on contributions from the 11th International Plant Sulfur Workshop held on 16–20 September 2018 in Conegliano, Italy, and their contributions in plant S research is highlighted below.

## 1. New Insights into the Effect of Sulfur Availability on Plant Functionality

Sulfur limitation leads to a reduction of seed yield, nutritional quality, seed viability, and vigour. It is known that S metabolism is involved in the control of germination *sensu stricto* and seedling establishment, but it is largely unknown how the germination and the first steps of plant growth are impacted in seeds when the plants are subjected to sulfate limitation. D’Hooghe et al. (2019) [2] focused on the impact of various S-limited conditions applied to mother plants on the germination indexes and the rate of viable seedlings in a spring oilseed rape cultivar (cv. Yudal), as well as on the sulfate uptake capacity during development of the seedling. When seeds were produced under severe S limitation, viable seedlings from such seeds presented a higher dry biomass and were able to enhance the sulfate uptake by roots and the S translocation to shoots, although the rate of viable seedlings was significantly reduced along with the germination vigour and perturbations of post-germinative events were observed.

When plants are exposed to S limitation, the sulfate assimilation pathway is upregulated at the expense of growth-promoting measures, whilst after cessation of the stress, the protective measures are deactivated, and growth is restored. Indeed, transcripts of S deficiency marker genes are rapidly degraded when starved plants are resupplied with sulfur, but it remains unclear, which enzymes are responsible for the degradation of transcripts during the recovery from starvation. In eukaryotes, mRNA decay is often initiated by the cleavage of poly(A) tails via deadenylases, and mutations in the poly(A) ribonuclease PARN have been linked to altered abiotic stress responses in *Arabidopsis thaliana*. Moreover, the subcellular localization of PARN is currently disputed, with studies reporting both nuclear and cytosolic localization. Armbruster et al. (2019) [3] investigated the role of PARN in the recovery from S starvation. Despite the presence of putative PARN-recruiting AU-rich elements in S-responsive transcripts, S-depleted PARN hypomorphic mutants were able to reset their transcriptome to prestarvation conditions just as readily as wildtype plants. PARN was detected in cytoplasmic speckles, thus reconciling the diverging views in literature by identifying two PARN splice variants whose predicted localization is in agreement with those observations.

In *Arabidopsis thaliana,* a disruption mutant of SULTR1;2, sel1-10, has been characterized with phenotypes such as plants grown under S deficiency. Although the effects of S deficiency on S metabolism have been well investigated in seedlings, no studies have been performed on mature *A. thaliana* plants. Morikawa-Ichinose et al. (2019) [4] analyzed the accumulation and distribution of S-containing compounds in different parts of mature sel1-10, as well as wildtype (WT) plants grown under long-day conditions. While the levels of sulfate, cysteine, and glutathione were almost similar between sel1-10 and WT, levels of glucosinolates (GSLs) differed depending on plant part. GSLs levels in the leaves and stems were generally lower in sel1-10 than those in WT; however, sel1-10 seeds maintained similar levels of aliphatic GSLs to those in WT plants. GSL accumulation in reproductive tissues was likely to be prioritized due to its role in S storage and plant defense even when sulfate supply in sel1-10 was limited.

Seeds of common bean (*Phaseolus vulgaris*) on the one hand constitute an excellent source of vegetable dietary protein, but on the other hand present suboptimal levels of both the essential S-containing amino acids, methionine, and cysteine, whilst they accumulate large amounts of the γ-glutamyl dipeptide of S-methylcysteine, and lower levels of free S-methylcysteine and S-methylhomoglutathione. Previous findings suggested two distinct metabolite pools: Free S-methylcysteine levels were high at the beginning of seed development and declined at mid-maturation, whilst there was a biphasic accumulation of γ-glutamyl-*S*-methylcysteine, at early cotyledon and maturation stages. A possible model involves the formation of S-methylcysteine by cysteine synthase from O-acetylserine and methanethiol, whereas the majority of γ-glutamyl-*S*-methylcysteine may arise from S-methylhomoglutathione. According to Saboori-Robat et al. (2019) [5] metabolite profiling during development and in genotypes differing in total S-methylcysteine accumulation, showed that γ-glutamyl-*S*-methylcysteine accounts for most of the total S-methylcysteine in mature seed. Profiling of transcripts for candidate biosynthetic genes indicated that BSAS4;1 expression was correlated with both the developmental timing and levels of free S-methylcysteine accumulated, while homoglutathione synthetase (hGS) expression was correlated with the levels of γ-glutamyl-*S*-methylcysteine. Analysis of S-methylated phytochelatins revealed only small amounts of homophytochelatin-2 with a single S-methylcysteine. The mitochondrial localization of phytochelatin-2 synthase predominant in seed and its spatial separation from S-methylhomoglutathione may explain the lack of significant accumulation of S-methylated phytochelatins.

Plants have developed sophisticated mechanisms for acquiring iron (Fe) from the soil and in the graminaceous species, a chelation strategy is in charge, in order to take up ferric Fe from the rhizosphere. The ferric Fe chelation-strategy components may also be present in the aerial plant parts. Saridis et al. (2019) [6] searched for possible roles of those components in maize leaves, therefore the expression patterns of ferric Fe chelation-strategy components were monitored in the leaves and roots of mycorrhizal and nonmycorrhizal S-deprived maize plants, both before and after sulfate supply. Altering of S supply was chosen due to the strong impact of S on iron homeostasis, whilst mycorrhizal symbiosis was chosen as a treatment that forces the plant to optimize its photosynthetic efficiency, in order to feed the fungus. The results suggested a role for the aforementioned components in ferric chelation and/or unloading from the xylem vessels to the aerial plant parts and it was proposed that the gene expression of the DMA exporter ZmTOM1 can be used as an early indicator for the establishment of a mycorrhizal symbiotic relationship in maize.

## 2. The Role of S Deficiency in Root System Functionality

Root hairs contribute to nutrient uptake from the root environment, but the contribution varies among nutrients. In *Arabidopsis thaliana*, the two high-affinity sulfate transporters SULTR1;1 and SULTR1;2, are responsible for sulfate uptake by roots. Their increased expression under S deficiency (−S) stimulates sulfate uptake. Inspired by the higher and lower expression, respectively, of SULTR1;1 in mutants with more (werwolf [wer]) and fewer (caprice [cpc]) root hairs, Kimura et al. (2019) [7] examined the contribution of root hairs to sulfate uptake. Its uptake rates were similar among plant lines under both S sufficiency and deficiency. Under S deficiency, the expression of SULTR1;1 and SULTR1;2 was negatively correlated with the number of root hairs. These results suggested that both SULTR expression and sulfate uptake rates induced by S deficiency were independent of the number of root hairs. A negative correlation between primary root lengths and number of root hairs, along with a greater number of root hairs was observed under S deficiency than under S sufficiency, thus suggesting that under both S sufficiency and deficiency, sulfate uptake was influenced by the root biomass rather than the number of root hairs.

Architecture of the plant root system changes drastically in response to the availability of macronutrients in the soil environment. Despite the importance of root S uptake in plant growth and reproduction, molecular mechanisms underlying root development in response to S availability have not been fully characterized. Dong et al. (2019) [8] reported on the signaling module composed of the CLAVATA3 (CLV3)/EMBRYO SURROUNDING REGION (CLE) peptide and CLAVATA1 (CLV1) leucine-rich repeat receptor kinase, which regulate lateral root (LR) development in *Arabidopsis thaliana* upon changes in S availability. The wildtype seedlings exposed to prolonged S deficiency showed a phenotype with low LR density, which was restored upon sulfate supply. In contrast, under prolonged S deficiency the clv1 mutant showed a higher daily increase rate of LR density relative to the wildtype, which was diminished to the wildtype level upon sulfate supply. CLE2 and CLE3 transcript levels decreased under S deficiency and through CLV1-mediated feedback regulations. It is suggested that under S-deficient conditions CLV1 directs a signal to inhibit LR development, and the levels of CLE peptide signals are adjusted in the course of LR development. The study demonstrated a fine-tuned mechanism for LR development coordinately regulated by CLE-CLV1 signaling and in response to changes in S availability.

## 3. Role of S in Plants Grown under Drought Conditions

Abscisic acid (ABA) is the canonical trigger for stomatal closure upon drought. Soil-drying is known to facilitate root-to-shoot transport of sulfate, whereas sulfate and sulfide have been independently shown to promote stomatal closure. For induction of stomatal closure, sulfate must be incorporated into cysteine, which triggers ABA biosynthesis by transcriptional activation of NCED3. Rajab et al. (2019) [9] applied reverse genetics to unravel if the canonical ABA signal transduction machinery is required for sulfate-induced stomata closure, and if cysteine biosynthesis is also mandatory for the induction of stomatal closure by sulfide. The importance of reactive oxygen species (ROS) production by the plasma membrane-localized NADPH oxidases, RBOHD, and RBOHF is documented, during the sulfate-induced stomatal closure. In agreement with the established role of ROS as the second messenger of ABA-signaling, the SnRK2-type kinase OST1 and the protein phosphatase ABI1 are essential for sulfate-induced stomata closure, whilst sulfide failed to close stomata in a cysteine-biosynthesis depleted mutant. The presented data support the hypothesis that the two mobile signals, sulfate and sulfide, induce stomatal closure by stimulating cysteine synthesis to trigger ABA production.

## 4. Sulfur Nutrition under Field Conditions

Determination of the S status is very important to detect S deficiency and to prevent losses of yield and seed quality. Etienne et al. (2018) [10] studied the possibility of using the ([Cl^−^]+[NO_3_^−^]+[PO_4_^3−^]):[SO_4_^2−^] ratio as an indicator of S nutrition in *Brassica napus* under field conditions, and whether this could be applied to other species. Different S and nitrogen (N) fertilization schemes were applied on a S-deficient field cultivated with oilseed rape. Mature leaves were harvested, and their anions and element contents were analyzed in order to evaluate the aforementioned S nutrition indicator, along with useful threshold values. Large sets of commercial varieties were used to test S deficiency scenarios. Under field conditions, the leaf ([Cl^−^]+[NO^3−^]+[PO_4_^3−^])/[SO_4_^2−^] ratio was increased by lowering S fertilization, indicating S deficiency. The usefulness of this ratio was also found for other species grown under controlled conditions. Furthermore, the ratio could be simplified by using the ratio based on the elemental concentrations ([Cl]+[P])/[S] determined by X-ray fluorescence (XRF). Based on the ratio quantified under field conditions, threshold values were determined and used for the clustering of commercial varieties within three groups: S-deficient, at risk of S deficiency, and S sufficiency. It is suggested that the elemental ([Cl]+[P])/[S] ratio can be used as an early and accurate diagnostic tool to manage S fertilization.

The demand to develop fertilizers with higher S use efficiency has been intensified over the last decade, since S deficiency in crops has become more widespread. Bouranis et al. (2018) [11] investigated whether fertilizers enriched with 2% elemental sulfur (ES) via a binding material of organic nature improve yield when compared to the corresponding conventional ones. Several fertilization schemes with or without incorporated ES were tested in various durum wheat varieties, cultivated in commercial fields. The Olsen-P content of each commercial field was found to be correlated with the corresponding relative change in the yields with a strong positive relationship and the content of 8 ppm of available soil phosphorus (P) was a turning point. At higher values the incorporation of ES in the fertilization scheme resulted in higher yield, while at lower values it resulted in lower yield, compared with the conventional one. The application of fertilizer mixtures containing the urease inhibitor N-(n-butyl) thiophosphoric triamide (NBPT), ES, and ammonium sulfate resulted in the highest relative yields. The yield followed a positive linear relationship with the number of heads per square meter. In this correlation, the Olsen-P content separated the results of the two groups of blocks, where the applied linear trend line in each group presented the same slope.

In order to assess and evaluate the effect of fertilizer granules enriched with ES on the dynamics of the rhizospheric bacteria in relation to the corresponding conventional ones, Bouranis et al. (2019) [12] performed comparative experiments. The enriched fertilization scheme was applied to a soil with Olsen-P at 7.8 mg kg^−1^, and to a control treatment where Olsen-P accounted for 16.8 mg kg^−1^ in the soil. Rhizospheric soil at various developmental stages of the crops was analyzed and the agronomic profile of the rhizospheric cultivable bacteria was characterized and monitored. Additionally, the dynamics of P, Fe, organic S, and organic N were studied in both the rhizosoil and in the aerial part of the plant during crop development. Both crops were characterized by a comparable dry mass accumulation per plant throughout development, while the yield of the ES enriched crop was 3.4% less compared to the control crop. The ES enriched crop’s aerial parts showed a transient higher P and Fe concentration, while its organic N and S concentrations followed the pattern of the control crop. The incorporation of ES into the conventional fertilizer increased the percentage of arylsulfatase (ARS)-producing bacteria in the total bacterial population, suggesting an enhanced release of sulfate from the soil’s organic S pool, which the plant could readily utilize. A large fraction of the population also possessed phosphate solubilization, and/or siderophore production, and/or ureolytic traits, thus improving the crop’s P, Fe, S, and N balance. It is proposed that the used ES substantially improved the quality of the rhizosoil at the available P limiting level by modulating the abundance of the bacterial communities in the rhizosphere and effectively enhancing the microbially mediated nutrient mobilization towards improved plant nutritional dynamics.

The aforementioned original research articles clearly illustrate the dynamic nature of the topic, presenting a good example of the current progress in both the basic and applied aspects in the field of plant S research.
